# Supplementary material for: Transcriptome Analysis of Intracellular Amastigotes of Clinical Leishmania infantum Lines from Therapeutic Failure Patients after Infection of Human Macrophages
Source: Microorganisms. 2022 Jun 27;10(7):1304. doi: 10.3390/microorganisms10071304 (PMC9324091; doi:10.3390/microorganisms10071304)
Supplement: Supplementary file 1 [file microorganisms-10-01304-s001.zip › microorganisms-1752337-supplementary.pdf]

microorganisms

Supplementary materials

Table S1. DEGs for GO-enriched terms for the biological process category in LEM2126 *L. infantum* line.

GO term	GO ID	DEGs		
		Up	Down	Hypothetical
Protein phosphorylation	GO:0006468	21	0	0
Phosphorylation	GO:0016310	21	0	0
Organic acid biosynthetic process	GO:0016053	6	2	0
Carboxylic acid biosynthetic process	GO:0046394	6	2	0
Regulation of protein phosphorylation	GO:0001932	4	0	0
Regulation of phosphorylation	GO:0042325	4	0	0
Phosphate-containing compound metabolic process	GO:0006796	24	1	0
Phosphorus metabolic process	GO:0006793	24	1	0
Fatty acid biosynthetic process	GO:0006633	5	0	0
Monocarboxylic acid biosynthetic process	GO:0072330	5	0	0
Regulation of phosphate metabolic process	GO:0019220	4	0	0
Regulation of protein modification process	GO:0031399	4	0	0
Regulation of phosphorus metabolic process	GO:0051174	4	0	0
Alpha-amino acid metabolic process	GO:1901605	4	2	0
Fatty acid metabolic process	GO:0006631	5	0	0
Cellular protein modification process	GO:0006464	23	0	0
Protein modification process	GO:0036211	23	0	0
Small molecule biosynthetic process	GO:0044283	6	2	0
Carboxylic acid metabolic process	GO:0019752	9	2	0
Regulation of kinase activity	GO:0043549	3	0	0
Oxoacid metabolic process	GO:0043436	9	2	0
Organic acid metabolic process	GO:0006082	9	2	0
Glutamine family amino acid metabolic process	GO:0009064	2	1	0
Regulation of transferase activity	GO:0051338	3	0	0
Macromolecule modification	GO:0043412	24	0	0
Alpha-amino acid catabolic process	GO:1901606	3	0	0
Sulfur amino acid metabolic process	GO:0000096	2	1	0
Cellular amino acid catabolic process	GO:0009063	3	0	0

Gene Ontology (GO) enrichment analysis of differentially expressed genes (DEGs) of different *L. infantum* lines after infection of THP-1 cells. The analysis was based on $\log_2FC \geq 1$ or $\log_2FC \leq 1$ and false discovery rates (FDRs) ≤ 0.05.

Table S2. DEGs for GO-enriched terms for the biological process category in LEM3323 *L. infantum* line.

GO term	GO ID	DEGs		
		Up	Down	Hypothetical
Protein phosphorylation	GO:0006468	2	28	0
Cellular protein modification process	GO:0006464	7	36	1
Protein modification process	GO:0036211	7	36	1
Macromolecule modification	GO:0043412	9	38	2
Phosphorylation	GO:0016310	2	28	0
Phosphate-containing compound metabolic process	GO:0006796	2	33	0
Phosphorus metabolic process	GO:0006793	2	33	0
Cell cycle	GO:0007049	2	8	1
Nuclear división	GO:0000280	2	2	1

Gene Ontology (GO) enrichment analysis of differentially expressed genes (DEGs) of different *L. infantum* lines after infection of THP-1 cells. The analysis was based on $\log_2FC \geq 1$ or $\log_2FC \leq 1$ and false discovery rates (FDRs) ≤ 0.05.

Table S3. DEGs for GO-enriched terms for the biological process category in LEM5159 *L. infantum* line.

GO term	GO ID	DEGs		
		Up	Down	Hypothetical
Transmembrane transport	GO:0055085	4	10	0
Intracellular signal transduction	GO:0035556	0	7	0
Signal transduction	GO:0007165	1	7	0
Signaling	GO:0023052	1	7	0
Cell communication	GO:0007154	1	7	0
Response to stimulus	GO:0050896	6	7	0
Second-messenger-mediated signaling	GO:0019932	0	3	0
Nucleotide biosynthetic process	GO:0009165	1	5	0
Nucleoside phosphate biosynthetic process	GO:1901293	1	5	0
Interspecies interaction between organisms	GO:0044419	0	4	1
Regulation of response to stimulus	GO:0048583	1	3	0
Nucleotide metabolic process	GO:0009117	1	6	0
Nucleoside phosphate metabolic process	GO:0006753	1	6	0
Symbiont process	GO:0044403	0	3	0
Interaction with host	GO:0051701	0	3	0
Nucleobase-containing small molecule metabolic process	GO:0055086	1	7	0
Aromatic compound biosynthetic process	GO:0019438	3	7	0
Regulation of intracellular signal transduction	GO:1902531	0	3	0
Heterocycle biosynthetic process	GO:0018130	3	7	0
Cellular response to stimulus	GO:0051716	3	7	0
Carbohydrate derivative transport	GO:1901264	0	3	0
Organic cyclic compound biosynthetic process	GO:1901362	3	7	0

Gene Ontology (GO) enrichment analysis of differentially expressed genes (DEGs) of different *L. infantum* lines after infection of THP-1 cells. The analysis was based on $log_2FC \geq 1$ or $log_2FC \leq 1$ and false discovery rates (FDRs) ≤ 0.05.

Table S4. DEGs for GO-enriched terms for the biological process category in LLM2165 *L. infantum* line.

GO term	GO ID	DEGs		
		Up	Down	Hypothetical
Nucleobase-containing small molecule metabolic process	GO:0055086	5	4	0
Glutathione metabolic process	GO:0006749	0	3	0
Glutamine family amino acid metabolic process	GO:0009064	3	0	0
Purine-containing compound biosynthetic process	GO:0072522	3	2	0
Organic cyclic compound biosynthetic process	GO:1901362	8	3	0
Purine ribonucleotide biosynthetic process	GO:0009152	2	2	0
Purine-containing compound metabolic process	GO:0072521	3	3	0
Ribonucleotide biosynthetic process	GO:0009260	2	2	0
Ribose phosphate biosynthetic process	GO:0046390	2	2	0
Cellular modified amino acid metabolic process	GO:0006575	0	3	0
Sulfur compound metabolic process	GO:0006790	2	3	0
Purine nucleotide biosynthetic process	GO:0006164	2	2	0

Gene Ontology (GO) enrichment analysis of differentially expressed genes (DEGs) of different *L. infantum* lines after infection of THP-1 cells. The analysis was based on $log_2FC \geq 1$ or $log_2FC \leq 1$ and false discovery rates (FDRs) ≤ 0.05.

Table S5. DEGs for GO-enriched terms for the biological process category in LLM2070 *L. infantum* line.

GO term	GO ID	DEGs		
		Up	Down	Hypothetical
Protein glycosylation	GO:0006486	6	1	0
Glycoprotein biosynthetic process	GO:0009101	6	1	0
Macromolecule glycosylation	GO:0043413	6	1	0
Glycoprotein metabolic process	GO:0009100	6	1	0
Glycosylation	GO:0070085	6	1	0
Cellular protein modification process	GO:0006464	26	3	0
Protein modification process	GO:0036211	26	3	0
Macromolecule modification	GO:0043412	28	4	0
Regulation of cell cycle	GO:0051726	6	0	1
Translational elongation	GO:0006414	3	2	0
Macromolecule methylation	GO:0043414	4	1	0
Cell cycle	GO:0007049	8	0	1

Gene Ontology (GO) enrichment analysis of differentially expressed genes (DEGs) of different *L. infantum* lines after infection of THP-1 cells. The analysis was based on $\log_2 FC \geq 1$ or $\log_2 FC \leq 1$ and false discovery rates (FDRs) ≤ 0.05.

Table S6. DEGs for GO-enriched terms for the biological process category in LLM2221 *L. infantum* line.

GO term	GO ID	DEGs		
		Up	Down	Hypothetical
Macromolecule modification	GO:0043412	7	21	2
Posttranscriptional regulation of gene expression	GO:0010608	0	5	0
Cellular protein modification process	GO:0006464	4	17	1
Protein modification process	GO:0036211	4	17	1
Alpha-amino acid metabolic process	GO:1901605	2	3	0
Negative regulation of gene expression	GO:0010629	0	4	0

Gene Ontology (GO) enrichment analysis of differentially expressed genes (DEGs) of different *L. infantum* lines after infection of THP-1 cells. The analysis was based on $\log_2 FC \geq 1$ or $\log_2 FC \leq 1$ and false discovery rates (FDRs) ≤ 0.05.
